# Macadamia Oil Supplementation Attenuates Inflammation and Adipocyte Hypertrophy in Obese Mice

**DOI:** 10.1155/2014/870634

**Published:** 2014-09-22

**Authors:** Edson A. Lima, Loreana S. Silveira, Laureane N. Masi, Amanda R. Crisma, Mariana R. Davanso, Gabriel I. G. Souza, Aline B. Santamarina, Renata G. Moreira, Amanda Roque Martins, Luis Gustavo O. de Sousa, Sandro M. Hirabara, Jose C. Rosa Neto

**Affiliations:** ^1^Departamento de Biologia Celular e do Desenvolvimento, Universidade de São Paulo, Avenida Lineu Prestes 1524, Cidade Universitária, 05508-000 São Paulo, SP, Brazil; ^2^Programa de Pós-Graduação em Ciência da Motricidade, Departamento de Educação Física, Universidade Estadual Paulista (UNESP), 13506-900 Rio Claro, SP, Brazil; ^3^Departamento de Fisiologia e Biofísica, Instituto de Ciências Biomédicas, Universidade de São Paulo, 05508-000 São Paulo, SP, Brazil; ^4^Departamento de Ciências Biológicas, Laboratório de Movimento Humano da Universidade São Judas Tadeu, 05503-001 São Paulo, SP, Brazil; ^5^Departamento de Fisiologia, Disciplina de Fisiologia da Nutrição, Universidade Federal de São Paulo, 04023-901 São Paulo, SP, Brazil; ^6^Departamento de Fisiologia Geral, Instituto de Biociências, Universidade de São Paulo, 05508-090 São Paulo, SP, Brazil; ^7^Programa de Pós-Graduação em Ciência do Movimento Humano, Instituto de Ciências da Atividade Física e Esporte, Universidade Cruzeiro do Sul, 01506-000 São Paulo, SP, Brazil

## Abstract

Excess of saturated fatty acids in the diet has been associated with obesity, leading to systemic disruption of insulin signaling, glucose intolerance, and inflammation. Macadamia oil administration has been shown to improve lipid profile in humans. We evaluated the effect of macadamia oil supplementation on insulin sensitivity, inflammation, lipid profile, and adipocyte size in high-fat diet (HF) induced obesity in mice. C57BL/6 male mice (8 weeks) were divided into four groups: (a) control diet (CD), (b) HF, (c) CD supplemented with macadamia oil by gavage at 2 g/Kg of body weight, three times per week, for 12 weeks (CD + MO), and (d) HF diet supplemented with macadamia oil (HF + MO). CD and HF mice were supplemented with water. HF mice showed hypercholesterolemia and decreased insulin sensitivity as also previously shown. HF induced inflammation in adipose tissue and peritoneal macrophages, as well as adipocyte hypertrophy. Macadamia oil supplementation attenuated hypertrophy of adipocytes and inflammation in the adipose tissue and macrophages.

## 1. Introduction

The role of a diet with a higher content of unsaturated fatty acids, in place or concomitant to a diet with high content of lipids, has been appointed as an effective strategy to control metabolic disorders [1]. Monounsaturated fatty acids (MUFA) rich diet has been reported to decrease plasma total cholesterol and LDL-cholesterol and increase HDL-cholesterol levels [2–5]. Moreover, when saturated fatty acids are replaced by MUFA in the diet of obese women, levels of inflammatory markers decrease, including IL-6 and visfatin in serum [6]. Macadamia nut oil is rich in monounsaturated fatty acids, containing approximately 65% of oleic acid (C18:1) and 18% palmitoleic acid (C16:1) of the total content of fatty acids [7]. Macadamia oil is the main source of palmitoleic acid in the human diet. Some studies have shown that diet rich in macadamia can improve the lipid profile [2, 8–10], but to date there is no studies on the effect of supplementation of macadamia oil on adipocyte hypertrophy and inflammation.

In 2008, Cao and colleagues [11] showed that mice deficient in lipid chaperones aP2 and mal1 present increased levels of palmitoleic acid in serum. Elevated levels of circulating palmitoleic acid restored sensitivity of insulin in liver and skeletal muscle, hepatosteatosis, and hyperglycemia, generated by high-fat diet. With this, the authors named this fatty acid as a lipokine, since palmitoleic acid has a hormonal-like effect [11].

The administration of high-fat diet in C57BL6 mice induces metabolic perturbations similar to those observed in humans. In fact, consumption of the high levels of saturated fatty acids is associated with overweight, visceral obesity, inflammation, dyslipidemia, and insulin resistance, in skeletal muscle, liver, and adipose tissue [12–17]. Saturated FFA promotes inflammation by interaction with toll-like receptor 4 (TLR4), activating NF*κ*B, JNK, and AP-1 pathways [18, 19].

A low grade inflammation is established with increase in plasma levels of IL-6, IL-1*β*, prostaglandins, TNF-α, and leptin and decrease in the production and secretion of adiponectin, IL-10, and IL-4 [20, 21]. The increase in local inflammation is potentiated by the recruitment of macrophages to adipose tissue and polarization of M2 macrophages (macrophages type 2) to M1 macrophages (macrophages type 1) [16, 22, 23].

The aim of our study was to evaluate the effect of macadamia oil supplementation, rich in MUFA (palmitoleic and oleic acids), on adipose tissue and peritoneal macrophages inflammation in mice fed a balanced diet or high-fat diet rich in saturated fatty acids. We measured glucose uptake (2-6 deoxyglucose uptake) and mRNA content of proteins (GLUT-4; IRS-1) involved in insulin signaling in soleus muscle. The contents of IL-10, IL-6, TNF-α, and IL-1*β* in peritoneal macrophages and adipose tissue were also determined. The adipocyte size was also evaluated.

## 2. Materials and Methods

### 2.1. Animals

All experiments were performed according to protocols approved by the Animal Care and Use Committee of the Institute of Biomedical Sciences, University of São Paulo. C57BL/6 male mice (8 weeks old) were used in this study. Animals were housed with light-dark cycle of 12-12 h and temperature of 23 ± 2°C. Animals were divided into four groups: (a) control diet (CD), (b) high-fat diet (HFD), (c) control diet supplemented with macadamia nut oil (Vital Âtman, Uchoa, SP, Brazil) (CD + MO), and (d) high-fat diet supplemented with macadamia oil (HF + MO). Control groups were run concomitantly. The oil composition is shown in Table 1. During the first 4 weeks preceding the induction of obesity by HFD, all groups were* ad libitum* fed a control diet (76% carbohydrates, 9% fat, and 15% proteins). Similar protocol has been used in our previous studies [24, 25]. CD + MO and HF + MO were supplemented by oral gavage at 2 g per Kg of body weight, three times per week, during 12 weeks. This dosage of oil was chosen based on previous studies from our group using different oils with no signs of hepatic toxicity [24]. CD and HF diet received water at the same dose.

### 2.2. Serum Parameters Analysis

Serum triacylglycerol, total cholesterol, LDL-cholesterol, and HDL-cholesterol were determined by colorimetric assays (Labtest Diagnostics, Lagoa Santa, MG, Brazil). Serum glucose and insulin were measured using LABTEST colorimetric assay and radioimmunoassay (Millipore, Billerica, MA, USA), respectively, as described by Masi et al. (2012) [24]. The HOMA index was determined by calculating fasting serum insulin (*μ*U/mL) × fasting plasma glucose (mmol L^−1^)/22.5. Leptin and adiponectin were measured using the protocol of the manufacturing R&D system.

### 2.3. GTT and ITT

Glucose tolerance test (GTT) and insulin tolerance test (ITT) were carried out in all groups after 6 h fasting at the end of the 10th and 11th weeks of treatment, respectively.

The methodologies used for GTT and ITT were similar to that described by Masi et al. (2012) [24].

### 2.4. Insulin Responsiveness in Incubated Soleus Muscle

Animals were euthanized on CO_2_ chamber and soleus muscles rapidly and carefully isolated and weighed (8–10 mg). This protocol was described in [24, 25].

### 2.5. Haematoxylin and Eosin Staining

Adipose samples were fixed in formalin and paraffin embedded. Sections were prepared (5 *μ*M) using Leica EG1150H Machine. Haematoxylin and Eosin (H&E) staining was conducted using Leica Autostainer XL and Leica CV5030. Sections were mounted using DPX media (Fisher Scientific, Ireland) and analyzed using Nikon 80i transmission light microscope.

### 2.6. Extraction of Fatty Acids from Gastrocnemius Muscle and Gas Chromatographic Analysis

Gastrocnemius muscle fragments (100 mg) were subjected to lipid extraction. For this, 0.5 mL chloroform/methanol (2 : 1; v/v) was added to 100 mg of gastrocnemius sample, well-vortexed and incubated at room temperature for 5 min. Additional volumes of 1.25 mL chloroform and 1.25 mL deionized H_2_O were then added, and finally, following vigorous homogenization for 3 min, samples were centrifuged at 1200 g for 5 min, at room temperature to obtain two phases: aqueous phase in the top and organic phase in the bottom containing. The organic phase was collected, dried, and suspended in isopropanol. Triglyceride content was then determined in the homogenate. After that, for fatty acid composition determination, gastrocnemius lipid extracts were dried using atmospheric N_2_ for evaporation of the solvent without fatty acid oxidation. The fractions of neutral and polar lipids were separated from these extracts by using a column chromatography. The polar (phospholipids) and neutral (triglycerides) fractions were methylated (for formation of methyl esters), using acetyl chloride and methanol. The methyl esters were analyzed in a gas chromatographer coupled to a flame ionizer detector (FID) (Varian GC 3900). Fatty acid composition was then determined by using standard mixtures of fatty acids with known retention times (Supelco, 37 Components).

For the analysis of fatty acids, a programmed chromatography was used with the characteristics described below. The reading was initiated at 170°C temperature for 1 minute and then a ramp of 2.5°C/min was employed to reach a final temperature of 220°C that was maintained for 5 min. The injector and detector were maintained at 250°C. We used the* CP wax 52 CB* column, with a 0.25 mm thickness, internal diameter of 0.25 mm, and 30 mm long, with hydrogen as the carrier gas.

### 2.7. Analysis of Inflammatory Parameters

#### 2.7.1. Adipokines Content Measurements

Mice were euthanized on CO_2_ chamber and retroperitoneal adipose tissue was rapidly collected. About 100 mg of retroperitoneal adipose tissue was used for the determination of TNF-α, IL-6, and IL-10 content. Adipose tissue was homogenized in RIPA buffer (0.625% Nonidet P-40, 0.625% sodium deoxycholate, 6.25 mM sodium phosphate, and 1 mM ethylenediaminetetraacetic acid at pH 7.4), containing 10 g/mL of a protease inhibitor cocktail (Sigma-Aldrich, St. Louis, MO, USA). Homogenates were centrifuged at 12.000 g for 10 min at 4°C, supernatant was collected, and protein concentration was determined using Bradford assay (Bio-Rad, Hercules, CA, USA). Bovine serum albumin was used as protein standard.

*Ex Vivo Adipose Tissue Culture*. Retroperitoneal adipose tissue explants (about 100 mg) were cultured in DMEM sterile medium (Gibco), containing 10% FBS, 2 mM glutamine, streptomycin, and penicillin for 24 h, at 37°C and 5% CO_2_, humidified air environment. Thereafter, medium culture was collected and used for the determination of IL-1*β* and IL-10, using ELISA assays (DuoSet kits, R&D System).

#### 2.7.2. Peritoneal Macrophage Isolation and Culture

Cytokine and nitric oxide (NO) production were evaluated in macrophages obtained by washing the peritoneal cavity with 6 mL RPMI culture medium (Gibco), containing 10% FBS and 4 mM glutamine. Macrophage-rich cultures (more than 90% of the cells were F4/80^+^) were obtained by incubating peritoneal cells in 24-well polystyrene culture plates for 2 h at 37°C in a 5% CO_2_, humidified air environment. Nonadherent cells were removed by washing with RPMI. Adherent cells were then incubated with 2.5 *μ*g/mL of LPS (*E. coli*, serotype 0111:B4, Sigma Chemical Company, USA) for 24 h [26]. Medium was collected for determination of IL-6, IL-10, IL-1B and TNF-α by ELISA and nitrite content by Griess method [27].

### 2.8. Quantitative RT-PCR

Total RNA from the gastrocnemius muscle was extracted with Trizol reagent (Invitrogen Life Technologies, Grand Island, NY, USA), following the method described by Chomczynski and Sacchi [28]. Reverse transcription to cDNA was performed using the high-capacity cDNA kit (Applied Biosystems, Foster, CA, USA). Gene expression was evaluated by real-time PCR [29], using Rotor Gene (Qiagen) and SYBR Green (Invitrogen Life Technologies) as fluorescent dye. Primer sequences are shown in Table 2. Quantification of gene expression was carried out using the RPL-19 gene as internal control, as previously described [30].

### 2.9. Statistical Analysis

Results are presented as mean ± S.D. All groups were compared by using two-way ANOVA followed by Bonferroni posttest. Significance level was set at *P* < 0.05.

## 3. Result

### 3.1. Characterization of the Experimental Model

#### 3.1.1. Body Composition

**Figure 1:**
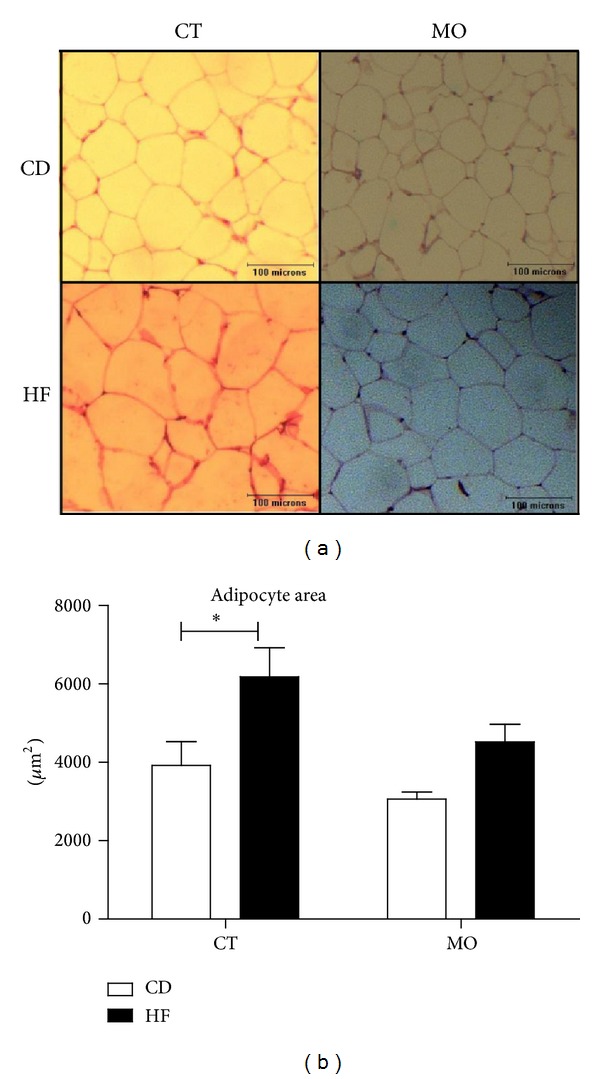
Effect of MO supplementation on adipose tissue histology. (a) Histological sections stained with H&E. (b) Area of adipocytes. CD = group of animals maintained on control diet; HF = group of animals fed high-fat diet; CD + MO = group of animals fed control diet supplemented with macadamia oil; HF + MO = group of animals fed high-fat diet supplemented with macadamia oil. The data are given as the means ± S.D. *P < 0.05 (*n* = 6).

**Figure 2:**
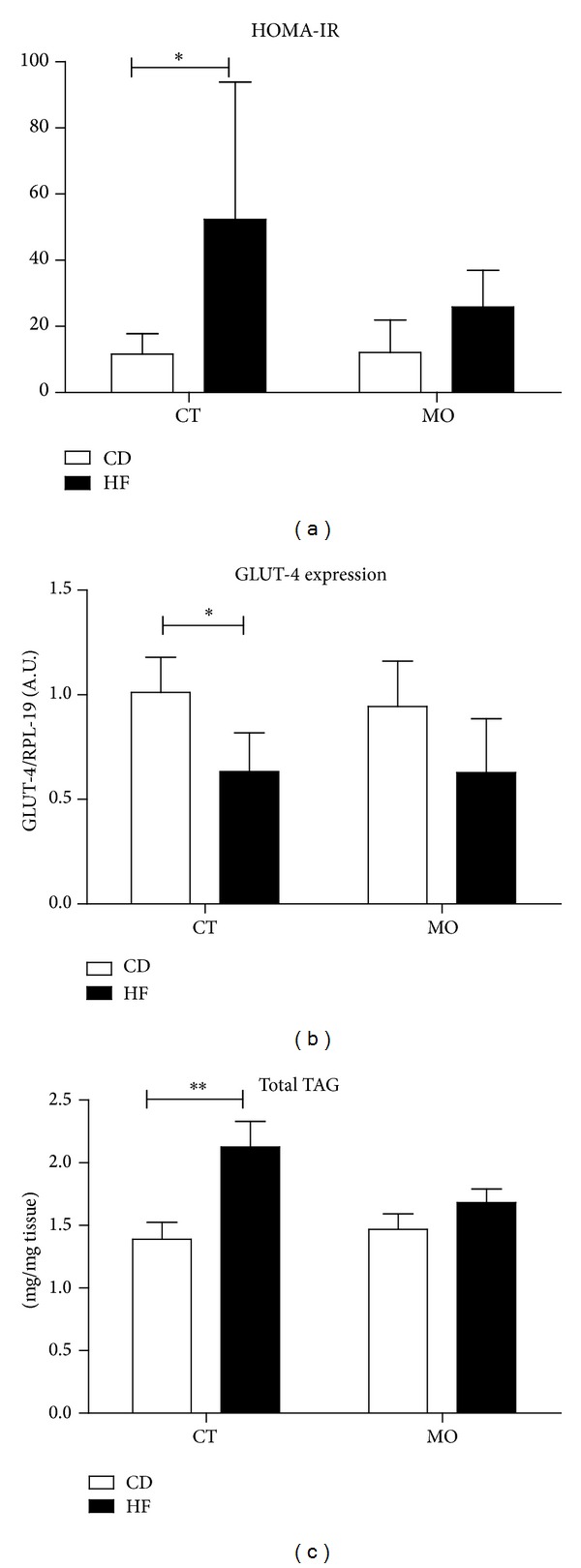
Insulin sensitivity and triacylglycerol content in skeletal muscle after 12 weeks. (a) HOMA-IR: homeostatic model assessment of insulin resistance; (b) GLUT-4 gene expression; (c) triacylglycerol content in gastrocnemius muscle. The data are given as the means ± S.D. In all experiments the animals were previously fasted for 6 hours. CD = group of animals maintained on control diet; HF = group of animals fed high-fat diet; CD + MO = group of animals fed control diet supplemented with macadamia oil; HF + MO = group of animals fed high-fat diet supplemented with macadamia oil. A.U. = arbitrary unit. *P < 0.05; **P < 0.01 (*n* = 6).

Mice fed high-fat diet showed increased body weight gain, hypercholesterolemia, and insulin resistance. These modifications were similar to those observed in our previous studies [24, 25]. Animals fed the high-fat diet (HF and HF + MO) for eight weeks showed increased (by 2-fold) body weight gain and visceral adiposity index as compared with CD and CD + MO (Table 3). The weights of the liver and the brown adipose tissue depot were not altered with diet or supplementation (Table 3). Although the visceral adiposity index of mice fed high-fat diet (HF and HF + MO) was greater than in animals that received control diet (CD and CD + MO), the HF group had an increase (by 1,62-fold) of adipocytes size compared to the control diet (Figures 1(a) and 1(b)), with statistical difference not evidenced in HF + MO group. No difference was evidenced by diet or supplementation in LDL-c, NEFA, and glycerol (data not shown). Moreover, the basal glycemia and *K*_itt_ were increased in both groups treated with high-fat diet (data not shown). Homa-IR index was increased in the HF group (by 3-fold) as compared to the other groups including the HF + MO (Figure 2(a)). This result suggests a beneficial effect of macadamia oil supplementation on insulin responsiveness in the HF group.

The peripheral insulin resistance was confirmed by glucose uptake in incubated* soleus* muscle (data not shown), as also shown previously [24, 25]. In addition, both groups treated with high-fat diet showed decrease in GLUT-4 mRNA expression (Figure 2(b)). The PGC-1, IRS-1, CPT-1 and Perilipin 5 mRNA expression were not modulated in our treatment. The HF group showed an increase in triacylglycerol content in gastrocnemius muscle, but this effect was blunted in HF + MO (Figure 2(c)). The fatty acid composition in neutral or polar lipid fractions remains unchanged regardless of the diet given and MO supplementation (see Table 1 in Supplementary Material available online at http://dx.doi.org/10.1155/2014/870634).

### 3.2. Macadamia Oil Supplementation Attenuates High-Fat Diet Induced Inflammation

**Figure 3:**
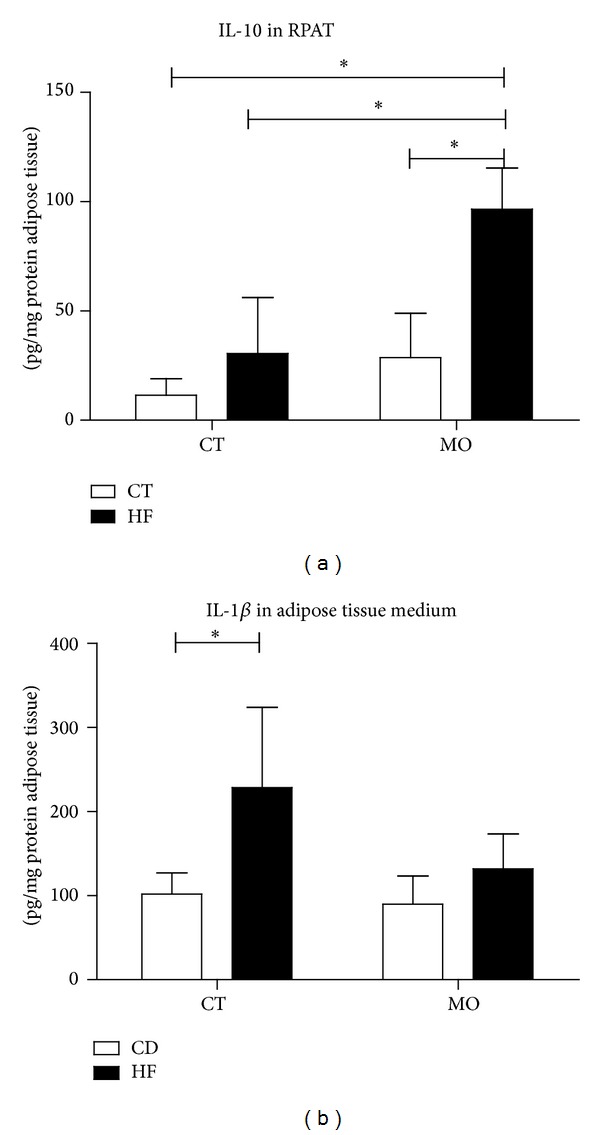
Inflammatory parameters in adipose tissue homogenate and adipose tissue explant incubation medium. IL10 content in adipose tissue homogenate (a) and IL1-*β* in the adipose tissue explant incubation medium, after 24 hours measured by ELISA (b). The animals received water or macadamia oil orally, 2 g/kg b.w., with or without association with a high-fat diet. CD = group of animals maintained on control diet; HF = group of animals fed high-fat diet; CD + MO = group of animals fed control diet supplemented with macadamia oil; HF + MO = group of animals fed high-fat diet supplemented with macadamia oil. The data are given as the means ± S.D. *P < 0.05 (*n* = 5-6).

The contents of the anti-inflammatory cytokine IL-10 were increased in the HF + MO group (approximately 4,09-fold) (Figure 3(a)), while IL-1b concentration in the medium of adipose tissue explants was increased in the HF group (Figure 3(b)).

**Figure 4:**
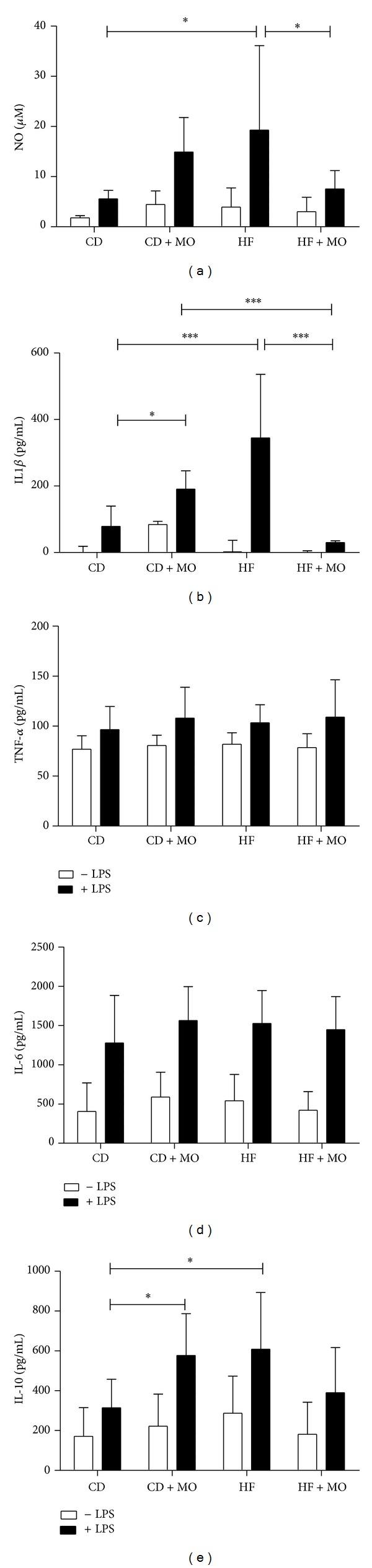
Nitric oxide and cytokine production by peritoneal macrophages. Peritoneal macrophages were collected and cultured for 24 h in the absence (white bars) or presence (black bars) of 2.5 *μ*g/mL LPS. Nitric oxide (a), IL1-*β* (b), TNF-α (c), IL-6 (d), and IL-10 (e) were measured. CD = control diet; HF = high-fat diet; CD + MO = control diet + macadamia oil; HF + MO = high-fat diet + macadamia oil. The data are given as the means ± S.D. *P < 0.05; ***P < 0.001 (*n* = 5-6).

When stimulated with LPS, macrophages from all groups showed increased IL-6 production (by 2.97-fold) (Figure 4(d)), whereas IL-10 and NO production were elevated in cells from the HF and CT + MO groups (2.39- and 4.08-fold compared to base line, resp.) (Figures 4(a) and 4(e)). No effect of LPS stimulation was observed on TNF-α production by macrophages from all groups (Figure 4(c)).

Moreover, macrophages from the HF group showed an increase (by 2,41-fold) of IL-1*β* production compared to unstimulated cells whereas the supplementation with MO abolished this elevation (Figure 4(d)). Similar results were found in NO production. MO attenuated nitrate production by LPS stimulation on macrophages from the HF group (Figure 4(a)). The production of IL-10 was decreased in the CT group compared to CT + MO and HF groups (by 1,88-fold) (Figure 4(e)). TNF-α and IL-6 production remained unchanged by diet or supplementation (Figures 4(c) and 4(d)).

No significant difference was found in serum levels of adiponectin after 12 weeks of treatment (data not shown). As expected, leptin concentration was increased (by 5.69-fold) in the groups fed the high-fat diet (data not shown). The significant difference between the CD + MO and the CD + CT groups suggests that MO can enhance circulant leptin.

## 4. Discussion

We showed herein that twelve weeks of macadamia oil supplementation attenuate the increase in inflammation and adipocyte hypertrophy in mice fed a high-fat diet that exhibit signs of the metabolic syndrome.

High consumption of fat, sucrose, and industrialized foods in association with sedentary lifestyle is the main contributor to obesity and its related comorbidities, including dyslipidemias, insulin resistance, and cardiovascular diseases; evidence has been accumulated that low grade inflammation plays a key role in the obesity induced comorbidities [11, 31–37].

The increase in adipocytes size was attenuated by macadamia oil treatment. The increase in adipocyte diameter has been associated with disturbances in cellular homeostasis, such as insulin resistance, inflammation, and hypoxia [38]. The prevalence of large adipocytes increases leptin production and secretion, as observed in our study [39]. The increase in leptin is associated with an elevation in low grade inflammation. Leptin is known to stimulate proinflammatory cytokines production in lymphocytes [40–42], monocytes [43], and macrophages [44].

Mice fed the HFD for 8 weeks exhibited increased IL-10 content in retroperitoneal adipose tissue. This result may be associated with the increase in peroxisome proliferator activated receptor- (PPAR-) gamma activity. This nuclear receptor increased the number of small adipocytes and raised the IL-10 [45, 46]. The increase of IL-10 content in adipose tissue leads to macrophage polarization (type 2) that is important for remodeling and tissue repair [47, 48]. Moreover, the increase in IL-10 content in adipocytes is associated with increased insulin sensitivity in adipose tissue [49, 50].

IL-1*β* strongly induces the inflammatory response in innate immune cells [51], via JNK and NF*κ*B pathway [52]. IL-1*β* is also a potent inductor of insulin resistance. This cytokine decreased insulin-stimulated glucose uptake via ERK activation [53]. Patients with high level of the circulating IL-1*β* are associated with greater risk on development of type 2 diabetes [54]. Adipose tissue and peritoneal macrophages are two sources of IL-1*β*, and macadamia oil supplementation was effective in decreasing the production of this cytokine in both. However, unexpectedly, the CDM showed an increased IL-1*β* production after LPS stimulation in peritoneal macrophages.

NO production is increased in LPS-stimulated macrophages being more pronounced in mice fed high-fat diet [24]. We demonstrated herein that the same pattern and the supplementation with macadamia oil prevented the production of NO by peritoneal macrophages from HF mice. Other bioactive compounds, such as epigallocatechin gallate and resveratrol [55], decrease NO production by macrophage inhibition through of MAP kinase, JNK, and NF*κ*B signaling [56].

In conclusion, macadamia oil supplementation attenuated inflammation and adipocyte hypertrophy in obese mice.

## Supplementary Material

The fatty acid composition in neutral or polar lipid fractions was unchanged in gastrocnemius muscle, regardless of the diet and macadamia oil supplementation.

## Figures and Tables

**Table 1:** Fatty acid composition of macadamia oil.

Fatty acid	%
C12:0 lauric acid	0.09
C14:0 myristic acid	0.82
C16:0 palmitic acid	8.45
C16:1n7 palmitoleic acid	19.11
C17:0 heptadecanoic acid	0.28
C16:2n4 9,12-hexadecadienoic acid	0.02
C16:3n4 6,9,12-hexadecatrienoic acid	0.06
C18:0 stearic acid	3.90
C18:1n9 oleic acid	56.35
C18:1n7 vaccenic acid	3.09
C18:2n6 linoleic acid (LA)	1.35
C18:3n3 linolenic acid (ALA)	0.12
C20:0 arachidic acid	2.79
C20:1n9 gondoic acid	2.18
C20:1n11 gadoleic acid	0.12
C22:0 behenic acid	0.75
C22:1n9 erucic acid	0.22
C22:5n3 eicosapentaenoic acid	0.30

SFA	16.08
MUFA	80.01
PUFA	1.83
PUFA n3	0.42
PUFA n6	1.35
n3/n6	0.31

SFA = saturated fatty acids, sum of C12:0, C14:0, C16:0, C17:0, C18:0, C20:0, and C22:0; MUFA = monounsaturated fatty acids, sum of C16:1, C18:1n7, C18:1n9, C20:1n9, C20:1n11, and C22:1n9; PUFA = polyunsaturated fatty acids, sum of C16:3n4, C18:2n6, C18:3n3, and C22:5n3; PUFA n3 = sum of C18:3n3 and C22:5n3; PUFA n6 = C18:2n6.

**Table 2:** Primer sequences of the genes studies for real-time PCR.

Primer name	Forward	Reverse
RPL-19	5-AGC CTG TGA CTG CCA TTC-3	5-ACC CTT CCT CTT CCC TAT GC-3
GLUT-4	5-CAT TCC CTG GTT CAT TGT GG-3	5-GAA GAC GTA AGG ACC CAT AGC-3
IRS-1	5-CTC AGT CCC AAC CAT AAC CAG-3	5-TCC AAA GGG CAC CGT ATT G-3
CPT-1	5-CCT CCG AAA AGC ACC AAA AC-3	5-GCT CCA GGG TTC AGA AAG TAC-3
PGC1-a	5-CAC CAA ACC CAC AGA AAA CAG-3	5-GGG TCA GAG GAA GAG ATA AAG TTG-3
Perilipin 5	5-CAT GAC TGA GGC TGA GCT AG-3	5-GAG TGT TCA TAG GCG AGA TGG-3

**Table 3:** Effect of high fat diet, with or without supplementation of macadamia oil, on obesity characteristics.

	CD	CD + MO	HF	HF + MO
Initial body weight (g)	24.26 ± 5.02	24.77 ± 3.37	24.2 ± 3.42	24.4 ± 4.41
Final body weight (g)	26.2 ± 1.99	26.82 ± 3.32	34.49 ± 6.96^∗#^	34.75 ± 4.96^∗#^
Liver weight (g)	1.17 ± 0.14	1.10 ± 0.23	1.33 ± 0.52	1.24 ± 0.26
Mesenteric adipose tissue weight (g)	0.32 ± 0.14	0.28 ± 0.12	0.55 ± 0.28^∗#^	0.52 ± 0.23^∗#^
Epididymal adipose tissue weight (g)	0.63 ± 0.14	0.73 ± 0.34	1.45 ± 0.60^∗#^	1.59 ± 0.70^∗#^
Retroperitoneal adipose tissue weight (g)	0.21 ± 0.06	0.24 ± 0.12	0.53 ± 0.22^∗#^	0.55 ± 0.17^∗#^
Adiposity index (g)	1.16 ± 0.24	1.25 ± 0.53	2.53 ± 1.01^∗#^	2.65 ± 1.01^∗#^
Brown adipose tissue weight (g)	0.104 ± 0.06	0.116 ± 0.03	0155 ± 0.06	0.128 ± 0.02

Values represent the means ± S.D. of the data obtained from analysis of 15 animals per group. *P < 0.001 versus CD; ^#^ *P* < 0.001 versus CD + MO.
