# Supplementary material for: Macadamia Oil Supplementation Attenuates Inflammation and Adipocyte Hypertrophy in Obese Mice
Source: Mediators Inflamm. 2014 Sep 22;2014:870634. doi: 10.1155/2014/870634 (PMC4190113; doi:10.1155/2014/870634)
Supplement: Supplementary file 1 — The fatty acid composition in neutral or polar lipid fractions was unchanged in gastrocnemius muscle, regardless of the diet and macadamia oil supplementation. [file 870634.f1.pdf]

Supplementary Material

Supplementary Figures

Table 1Sup. Polar and neutral fatty acid composition in gastrocnemius muscle after 12 weeks of treatment.

Table1Sup.

Polar Fatty Acids	CD	CD + MO	HF	HF + MO
C16	19.39 ± 1.2	20.55 ± 2.22	20.51 ± 4.03	20.76 ± 3.35
C16 n7	0.71 ± 0.9	0.71 ± 0.29	0.76 ± 0.21	0.56 ± 0.03
C18:0	10.89 ± 2.7	11.27 ± 0.23	10.89 ± 5.71	10.30 ± 3.76
C18:1 n9	32.87 ± 9.97	31.90 ±4.9	30.73 ± 15.89	31.77 ± 7.56
C18:1 n7	4.07 ± 0.34	3.77 ± 0.27	3.22 ± 0.48	3.22 ± 0.47
C18 n3	0.21 ± 0.08	0.23 ± 0.05	0.45 ±0.14	0.37 ± 0.13
C20-22 n3	7 ± 3.12	7.8 ± 0.72	8.64 ± 7.32	7.84 ± 2.84
C18/20-22 n3	0.04 ± 0.04	0.03 ± 0.01	0.08 ± 0.05	0.05 ± 0.01
C18 n6	8.01 ± 0.35	7.49 ± 3.07	11.08 ± 1.98	12.27± 1.63
C20-22 n6	12.26 ± 5.49	11.79 ± 2.38	9.24 ± 6.88	8.28 ± 3.69
C18/20-22 n6	0.81 ± 0.52	0.62 ± 0.15	1.83 ± 1.32	1.61 ± 0.46
SFA	33.15 ± 2.41	34.7 ± 2.62	34.11 ± 4.48	34.22 ± 0.06
MUFA	38.38 ± 10.57	37.21 ± 4.84	35.53 ± 17	36.34 ± 8.1
PUFA	28.4 ± 8.15	27.97 ± 5.77	30.25 ± 14.05	29.36 ± 8.12
PUFA n3	7.21 ± 3.04	8.03 ± 0.69	9.09 ± 7.31	8.21 ± 2.96
PUFA n6	20.26 ± 5.14	19.28 ± 5.26	20.31 ± 6.7	20.55 ± 5.23
n3/n6	0.35 ± 0.08	0.44 ± 0.11	0.4 ± 0.22	0.4 ± 0.08

Neutral Fatty Acids	CD	CD + MO	HF	HF + MO
C16	23.61 ± 0.77	21.60 ± 2.12	18.65 ± 0.99	20.03 ± 0.61
C16 n7	7.96 ±10.49	5.72 ± 7.12	0.51 ± 0.01	0.51 ± 0.01
C18:0	2.56 ± 0.64	2.43 ± 0.02	3.41 ± 0.41	3.03 ± 0.16
C18:1 n9	48 ± 5.24	49.49 ± 8.47	52.65 ± 0.61	47.42 ± 4.19
C18:1 n7	3.98 ± 0.05	4.06 ± 0.62	2.99 ± 0.09	2.91 ± 0.11
C18 n3	0.14 ± 0.12	0.32 ± 0.12	0.50 ± 0.06	0.44 ± 0.24
C20-22 n3	0.32 ± 0.13	0.26 ± 0.10	0.23 ± 0.08	1.99 ± 2.50
C18/20-22 n3	0.56 ± 0.61	1.26 ± 0.03	2.30 ± 0.59	1.42 ± 1.91
C18 n6	7.04± 1.37	9.46 ± 1.25	17.40 ± 0.27	13.10±6.37
C20-22 n6	2.84 ± 2.18	2.94 ± 3.17	1.01 ± 0.18	5.17 ± 4.0
C18/20-22 n6	3.25 ± 2.02	7.13 ± 7.27	17.61 ± 3.35	5.47 ± 6.32
SFA	28.23 ± 1.24	25.89 ± 2.47	23.39 ± 0.58	25.11 ± 0.35

MUFA	60.94 ± 4.57	60.60 ± 2.55	57.33 ± 0.62	51.80 ± 3.52
PUFA	10.72 ± 3.37	13.39 ± 5.01	19.22 ± 0.04	22.97 ± 3.13
PUFA n3	0.46 ± 0.01	0.58 ± 0.22	0.73 ± 0.13	2.43 ± 2.26
PUFA n6	9.88 ± 3.55	12.41 ± 4.42	18.40 ± 0.10	18.27 ± 1.57
n3/n6	0.05 ± 0.02	0.05 ± 0.01	0.04 ± 0.01	0.14 ± 0.13

SFA= Satured Fatty Acid, sum of C14:0, C15:0, C15:0iso, C 16:0, C17:0, C17:0 anteiso, C18:0, C18:0 iso, C18:0 anteiso, C20:0, C20:0 iso, C22:0 and C24:0. MUFA = Monounsaturated Fatty Acid, sum of C14:1 cis, C16:1 n7, C18:1 n9,C18:1 n7, C20:1 n9, C22:1. PUFA= Polyunsaturated Fatty Acid, sum of C16:3 n4, C18:3 n6, C18:3 n4, C18:3 n3, C18:4 n3, C20:2 n6, C20:3 n6, C20:4 n6, C22:5 n6, C22:5 n3 and C22:6 n3. PUFA n3 = sum of C18:3n3, 18:4 n3, C20:4 n3, C20:5, C22:5 n3 and C22:6 n3. PUFA n6 = sum of C18:2 n6c, C20:2 n6, C20:4 n6, C22:2 n6, C22:4 n6, C22:5 n6. CD = group of animals treated with control diet supplemented with water; HF= group of animals treated with High Fat Diet supplemented with water; CD + MO = group of animals treated with control diet supplemented with Macadamia Oil; HF + MO = HF= group of animals treated with High Fat Diet supplemented with Macadamia Oil. Values represent the mean and ± SD of the data obtained from analysis of 3 animals per group.
